# Season of delivery and risk of venous thromboembolism during hospitalization among pregnant women

**DOI:** 10.3389/fpubh.2023.1272149

**Published:** 2023-11-08

**Authors:** Qian Li, Hongfei Wang, Huafang Wang, Jun Deng, Zhipeng Cheng, Wenyi Lin, Ruiqi Zhu, Shi Chen, Jinrong Guo, Huarong Li, Yong Chen, Xiaowei Yuan, Shulan Dai, Yan Tian, Yanyan Xu, Ping Wu, Fan Zhang, Xiaojiang Wang, Liang V. Tang, Yu Hu

**Affiliations:** ^1^Institute of Hematology, Union Hospital, Tongji Medical College, Huazhong University of Science and Technology, Wuhan, Hubei, China; ^2^Department of Cardiovascular Surgery, Union Hospital, Tongji Medical College, Huazhong University of Science and Technology, Wuhan, Hubei, China; ^3^Department of Biobank, Union Hospital, Tongji Medical College, Huazhong University of Science and Technology, Wuhan, Hubei, China; ^4^Department of Medical Records Management and Statistics, Union Hospital, Tongji Medical College, Huazhong University of Science and Technology, Wuhan, Hubei, China; ^5^Department of Integrated Traditional Chinese and Western Medicine, Union Hospital, Tongji Medical College, Huazhong University of Science and Technology, Wuhan, Hubei, China; ^6^Department of Obstetrics and Gynecology, Jingshan Union Hospital, Union Hospital, Huazhong University of Science and Technology, Jingshan, Hubei, China; ^7^Department of Medical Services Division, People’s Hospital of Dongxihu District Wuhan City and Union Dongxihu Hospital, Huazhong University of Science and Technology, Wuhan, Hubei, China; ^8^Department of Obstetrics and Gynecology, People’s Hospital of Dongxihu District Wuhan City and Union Dongxihu Hospital, Huazhong University of Science and Technology, Wuhan, Hubei, China; ^9^Department of Obstetrics and Gynecology, Central Hospital of Hefeng County, Enshi, Hubei, China; ^10^Department of Neurology, Central Hospital of Hefeng County, Enshi, Hubei, China; ^11^Department of Obstetrics and Gynecology, The Sixth General Hospital of Hubei Province, Wuhan, Hubei, China; ^12^Department of Respiratory and Critical Care Medicine, The Sixth General Hospital of Hubei Province, Wuhan, Hubei, China

**Keywords:** GDM, IVF, pregnant women, season, venous thromboembolism

## Abstract

**Background:**

Seasons were found to be related to the occurrences of venous thromboembolism (VTE) in hospitalized patients. No previous study has explored whether seasons were associated with VTE risk in pregnant women. This study aimed to investigate the relationships between the season of delivery and VTE risk during hospitalization among pregnant women.

**Methods:**

This is a multi-center retrospective cohort study of pregnant women. Participants were those who delivered at seven designated sites in Hubei Province, China, during the period from January 2017 to December 2022. They were categorized according to their season/month of delivery. Information on new-onset VTE during hospitalization was followed.

**Results:**

Approximately 0.28% (104/37,778) of the pregnant women developed new-onset VTE during hospitalization for delivery. After adjustment, compared with participants in the spring group, participants in the summer, autumn, and winter groups had an increased risk of VTE during hospitalization. The ORs were 2.59 [1.30, 5.15], 2.83 [1.43, 5.60], and 2.35 [1.17, 4.75] for the summer, autumn, and winter groups, respectively. Pregnant women in the combined group (summer + autumn + winter) had an increased risk of VTE during hospitalization than those in the spring group (OR, 2.59 [1.39, 4.85]). By restricting the analyses among pregnant women without *in vitro* fertilization, gestational diabetes mellitus, and preterm, the results still remained robust. Compared with participants who delivered in March, April, and May, participants who delivered in June, July, September, November, December, and February had a higher risk of VTE during hospitalization.

**Conclusion:**

This study demonstrated that pregnant women who delivered in summer, autumn, and winter had an increased VTE risk during hospitalization compared with those who delivered in spring.

## Introduction

Venous thromboembolism (VTE), which includes deep vein thrombosis (DVT) and pulmonary embolism (PE), is a condition that occurs when a blood clot forms in a vein. During pregnancy, pregnant women have an approximately 4-fold increased risk of VTE than non-pregnant women due to venous stasis, pelvic vein compression by the uterine, and hypercoagulability. The incidences of VTE in pregnant women ranged from 0.82 to 19.97 per 10,000 deliveries in various countries and regions worldwide (1, 2). A meta-analysis of 20 studies reported that the pooled overall incidence of pregnancy-related VTE was 12 per 10,000 deliveries (3). VTE is a leading cause of maternal mortality and morbidity, posing a great threat to the health of mothers worldwide. Therefore, identifying more potential high-risk factors and strengthening VTE risk assessment and thromboprophylaxis among high-risk populations is of vital importance in the early prevention of VTE among pregnant women (4).

The known risk factors for pregnancy-related VTE included a personal past medical history of VTE, cesarean section delivery, preeclampsia, assisted reproduction methods, and other risk factors such as maternal age, parity, multiple pregnancies, gestational diabetes mellitus (GDM), and smoking (5–7). Meteorological conditions such as seasonal changes were found to have an important influence on the development of cardiovascular diseases (CVDs), leading to disorders of fibrinolysis, coagulation, and arterial blood pressure. Using data from 19 countries with seasonal variation, mortality from overall CVD and non-CVD/non-cancer was found to show a seasonal pattern (peak to nadir difference), being higher in winter than in summer (8).

Some previous studies have found that the occurrences of VTE/DVT/PE in hospitalized patients may be related to the seasons. Most studies found a high incidence of VTE/DVT/PE in winter, (9–17). some in autumn, (17–19) and a few in summer (20) and spring (21, 22). There was also one study reporting non-seasonal occurrences of VTE among hospitalized patients (23). The studies mentioned above have relatively small sample sizes (*n* < 2,800) and are mostly from a single center. Only three prior studies were performed in the Chinese population, and the conclusions were not consistent [with two being highest in winter (13, 14) and one being non-seasonal (23)]. At present, there is no prior report on the seasonal occurrences of VTE in pregnant women. Therefore, there is an urgent need for a large multi-center cohort of pregnant women to investigate the seasonality of VTE in pregnant women. Our hypothesis was that the season of delivery might be associated with VTE among pregnant women.

Based on a retrospective cohort of pregnant women in seven designated sites in Hubei Province, China, this study aimed to explore the relationships between the season of delivery and new-onset VTE during hospitalization.

## Materials and methods

### Study design

The participants came from a multi-center retrospective pregnant woman cohort in Hubei Province, China. The participants were pregnant women who were discharged after delivery from seven sites in Hubei Province, China, between January 2017 and December 2022. The pregnant women were enrolled at admission for delivery, and their delivery and health information during hospitalization were followed.

### Participants in this study

This study excluded pregnant women with previous VTE before admission or medication thromboprophylaxis within 2 weeks of enrollment. This study was approved by the ethics committee of Tongji Medical College affiliated with the Huazhong University of Science and Technology (No. [2015] S014) according to the principles of the Declaration of Helsinki. Informed consent was not required as the information was retrieved from the medical records retrospectively.

### Data collection

Information on demographics, lifestyle, history of reproduction, disease, medicine use, and diagnoses was collected at enrollment for delivery by doctors. Information on delivery and diagnoses at discharge was collected at discharge. Data on diagnoses at admission/discharge were retrieved from the electronic medical records and double-checked by two investigators (Qian Li and Hongfei Wang). To avoid diagnostic uncertainty, a third investigator (Liang V Tang) would join the discussion if any difference existed in the interpretation of the data.

### Definitions of seasons

The pregnant women in this study were categorized into four groups according to the season of delivery. Standard dates were used in the classification of seasons in each year, as recommended by Anar et al. (19). Spring was defined from 1 March to 31 May, summer was defined from 1 June to 31 August, autumn was defined from 1 September to 30 November, and winter was defined from 1 December 1 to 28/29 February (depending on whether or not it was a leap year) (19).

### Screening and diagnosis of VTE

Pregnant women who had new-onset VTE (DVT and/or PE) during hospitalization were defined as VTE cases. The diagnosis of DVT was made when there were standard clinical symptoms, and the findings of venography or ultrasonography were in agreement with DVT (24).^.^

PE was diagnosed if pregnant women had clinical symptoms together with the supportive findings of pulmonary angiography, a computed tomography, or a ventilation-perfusion lung scan (25). In the present study, only pregnant women with a definite VTE (DVT and/or PE) diagnosis at discharge (no history of VTE at admission or medication thromboprophylaxis within 2 weeks at admission) were defined as VTE cases; participants with superficial phlebitis were not considered VTE cases.

### Assessment of covariates

Covariates of maternal age at delivery (continuous), *in vitro* fertilization (IVF) pregnancy (yes/no), multiple pregnancies (yes/no), primipara (yes/no), habit of drinking (yes/no), habit of smoking (yes/no), history of diabetes (yes/no), gestational diabetes mellitus (GDM) (yes/no), preeclampsia (yes/no), preterm (yes/no), postpartum hemorrhage (yes/no), and delivery mode (cesarean/natural delivery) were considered potential confounders. IVF pregnancy was defined as the fertilization of an egg by a spermatozoon with assisted reproduction methods outside the body. The participants were asked whether they had the habit of smoking or drinking before and/or during pregnancy. According to the recommendation by the American Diabetes Association, GDM was diagnosed if the fasting, 1-h post-load, and 2-h post-load blood glucose levels at the oral glucose tolerance test reached any of the cutoff values of 5.1, 10.0, and 8.5 mmol/L (26). If pregnant women had new-onset gestational hypertension (BP ≥140/90 mm Hg) and proteinuria (>300 mg/24 h) after 20 weeks, preeclampsia would be diagnosed (27). Deliveries before 37 weeks of pregnancy were defined as preterm.

### Statistical analysis

In this study, maternal age at delivery was shown as a continuous covariate, expressed as mean ± standard deviation. The remaining covariates were categorical, expressed as n (%). The difference in maternal age at delivery among the groups was calculated by the *t*-test or one-way ANOVA. The variance of categorical covariates among the groups was calculated by the chi-square test. Logistic regression models were utilized to calculate the odds ratios (ORs) and 95% confidence intervals (CIs) for VTE during hospitalization according to the season of delivery (reference group: spring). This study reported the risk of VTE during hospitalization among pregnant women in the summer, autumn, and winter groups and the combined (summer + autumn + winter) group compared with those who delivered in spring. Demographic, disease history, lifestyle, disease, and birth outcomes were found to be risk factors for VTE postpartum (5–7). In this study, model 1 was adjusted for maternal age at delivery, IVF pregnancy, multiple pregnancies, and primipara. Model 2 was adjusted for habit of drinking, habit of smoking, history of diabetes, GDM, preeclampsia, preterm, postpartum hemorrhage, delivery mode, and all the covariates in model 1. The propensity score matching method (1:1) was applied to balance differences in the distributions of demographic characteristics, with the grouping variable as the dependent variable (delivered in spring season = 1, delivered in non-spring season = 0). A total of 9,188 accurate matching pairs were obtained for analyses. Sensitivity analyses of links between the season of delivery and VTE during hospitalization were conducted among pregnant women without IVF, GDM, and preterm using logistic regression models. This study explored the relationships between the month of delivery and VTE during hospitalization with logistic regression models (reference group: March + April + May). SAS version 9.4 and R version 4.2.1 were employed to analyze the data. A two-sided *p*-value of <0.05 was considered statistically significant.

## Results

### Study population

The study was conducted among the 37,908 pregnant women who delivered at seven sites in Hubei Province, China, between January 2017 and December 2022. This study excluded 130 pregnant women with previous VTE or medication thromboprophylaxis within 2 weeks of admission. A total of 37,778 pregnant women were included in the final analyses (Supplementary Figure S1). The pregnant women were categorized into four groups according to the season of delivery. The sample sizes for the spring, summer, autumn, and winter groups were 9,191 (24.3%), 9,808 (26.0%), 9,550 (25.3%), and 9,229 (24.4%), respectively.

### Characteristics of the pregnant women

Table 1 presents the social-demographic, reproductive, lifestyle, and other relevant characteristics of the pregnant women according to the season of delivery. Among the 37,778 pregnant women, the mean value of maternal age at delivery was 29.9 ± 4.6 years. The rates of IVF pregnancy, GDM, and preterm were significantly different in participants who delivered in various seasons (*p* < 0.05, Table 1). Other basic characteristics of the pregnant women who delivered in different seasons were similar. The basic characteristics were similar in pregnant women included and those excluded for previous VTE or medication thromboprophylaxis (Supplementary Table S1).

**Table 1:** Characteristics of the participants according to the season of delivery (*n* = 37,778).

Characteristics	Total (*n* = 37,778)	Season of delivery				
		Spring (*n* = 9,191)	Summer (*n* = 9,808)	Autumn (*n* = 9,550)	Winter (*n* = 9,229)	*p*-value
n/N (%)	100.0	24.3	26.0	25.3	24.4	
Maternal age at delivery (years)	29.9 ± 4.6	29.9 ± 4.6	30.0 ± 4.6	29.9 ± 4.6	29.9 ± 4.6	0.458
IVF pregnancy (%)	1712 (4.5)	411 (4.5)	506 (5.2)	403 (4.2)	392 (4.2)	0.005*
Multiple pregnancies (%)	934 (2.5)	242 (2.6)	251 (2.6)	215 (2.3)	226 (2.4)	0.355
Primipara (%)	23,562 (62.4)	5,788 (63.0)	6,178 (63.0)	5,883 (61.6)	5,713 (61.9)	0.098
Habit of drinking (%)	21 (0.06)	4 (0.04)	6 (0.01)	5 (0.1)	6 (0.1)	0.927
Habit of smoking (%)	126 (0.3)	35 (0.4)	23 (0.2)	27 (0.3)	41 (0.4)	0.054
History of diabetes (%)	84 (0.2)	21 (0.2)	22 (0.2)	21 (0.2)	20 (0.2)	0.998
GDM (%)	4,533 (12.0)	961 (10.5)	1,225 (12.5)	1,287 (13.5)	1,060 (11.5)	<0.001*
Preeclampsia (%)	1,094 (2.9)	263 (2.9)	271 (2.8)	271 (2.8)	289 (3.1)	0.459
Preterm (%)	2033 (5.4)	461 (5.0)	612 (6.2)	530 (5.5)	430 (4.7)	<0.001*

GDM, gestational diabetes mellitus; IVF, in vitro fertilization.

The comparison of “maternal age at delivery” among the four groups was performed with one-way ANOVA. The comparison of categorical variables among the four groups was performed with the chi-square test.

*p < 0.05.

### Associations between the season of delivery and VTE during hospitalization

**Figure 1:**
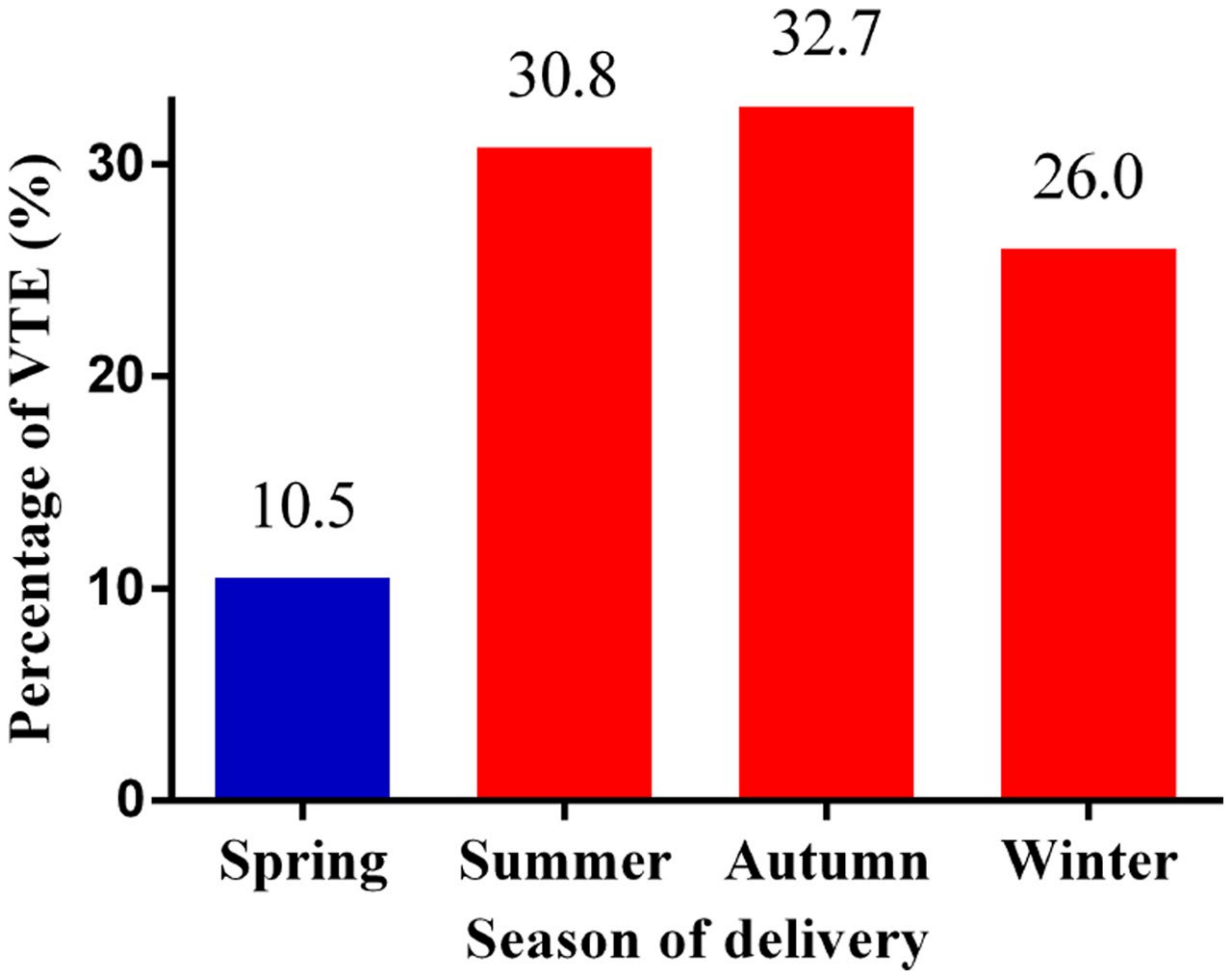
Percentages of VTE events according to the season of delivery. VTE: venous thromboembolism.

In this study, 104 (0.28%, 104/37778) pregnant women developed new-onset VTE during hospitalization. As shown in Figure 1, 10.5% (11/104), 30.8% (32/104), 32.7% (34/104), and 26.0% (27/104) of the VTE events occurred in spring, summer, autumn, and winter, respectively.

Pregnant women who delivered in summer (OR 2.73 [1.38, 5.42]), autumn (OR 2.98 [1.51, 5.89]), and winter (OR 2.45 [1.21, 4.94]) had an increased risk of VTE during hospitalization compared with those who delivered in spring. This study also suggested that pregnant women in the combined group (summer + autumn + winter) had a higher risk of VTE during hospitalization than those in the spring group (OR 2.72 [1.46, 5.09]). In model 2, compared with participants in the spring group, participants in the summer, autumn, and winter groups and the combined (summer + autumn + winter) group had an increased risk of VTE during hospitalization for delivery. The ORs were 2.59 [1.30, 5.15], 2.83 [1.43, 5.60], 2.35 [1.17, 4.75], and 2.59 [1.39, 4.85] for the summer, autumn, and winter groups and the combined group, respectively (Table 2). To eliminate the effects of demographic distribution differences on the results, 9,188 exact matches were obtained by propensity score matching (Supplementary Table S3). There were no significant differences in demographic distributions between the two newly matched groups (all *p* > 0.05; Supplementary Table S3). Compared with participants in the spring group, participants in the non-spring groups had an increased risk of VTE during hospitalization for delivery (OR 2.30 [1.13, 4.69]) (Supplementary Table S4).

**Table 2:** Adjusted ORs and 95% CIs for the season of delivery and VTE risk during hospitalization (*n* = 37,778).

	Season of delivery				
	Spring (*n* = 9,191)	Summer (*n* = 9,808)	Autumn (*n* = 9,550)	Winter (*n* = 9,229)	Combined (summer + autumn + winter)^a^ (*n* = 28,587)
No. of VTE (%)	11 (0.12%)	32 (0.33%)	34 (0.36%)	27 (0.29%)	93 (0.33%)
Unadjusted	1	2.73 (1.38, 5.42)	2.98 (1.51, 5.89)	2.45 (1.21, 4.94)	2.72 (1.46, 5.09)
Model 1	1	2.68 (1.35, 5.32)	2.98 (1.51, 5.88)	2.44 (1.21, 4.92)	2.70 (1.44, 5.05)
Model 2	1	2.59 (1.30, 5.15)	2.83 (1.43, 5.60)	2.35 (1.17, 4.75)	2.59 (1.39, 4.85)

CI, confidence interval; OR: odds ratio; VTE, venous thromboembolism.

Model 1 adjusted for maternal age at delivery, *in vitro* fertilization pregnancy, multiple pregnancies, and primipara.

Model 2 adjusted for habit of drinking, habit of smoking, history of diabetes, gestational diabetes mellitus, preeclampsia, preterm, postpartum hemorrhage, delivery mode, and covariates included in model 1.

a The reference group is spring.

### Sensitivity analyses

Restricting the analyses among pregnant women with non-IVF pregnancy, after adjustment, pregnant women in summer, autumn, and winter groups and in the combined (summer + autumn + winter) group had a higher risk of VTE during hospitalization than those in the spring group. The ORs were 2.63 [1.28, 5.40], 2.92 [1.43, 5.95], 2.21 [1.05, 4.65], and 2.59 [1.34, 5.00] for the summer, autumn, and winter groups and the combined group, respectively (Supplementary Table S5).

We repeated the analyses among pregnant women with non-GDM, and the associations between the season of delivery and VTE during hospitalization were consistent. In model 2, the ORs were 2.74 [1.23, 6.11], 3.09 [1.39, 6.85], 2.36 [1.03, 5.39], and 2.73 [1.31, 5.68] for the summer, autumn, and winter groups and the combined group, respectively (Supplementary Table S6).

We also performed sensitivity analyses among pregnant women without preterm. The increased risk of VTE during hospitalization in the summer (OR 3.66 [1.60, 8.40]), autumn (OR 4.12 [1.81, 9.38]), and winter (OR 3.68 [1.60, 8.47]) groups and the combined (OR 3.82 [1.77, 8.27]) group was consistently shown (Supplementary Table S7). In 2020, China’s Hubei Province suffered a sudden attack of the coronavirus disease 2019. This study excluded participants who enrolled in 2020 for sensitivity analyses and still found consistent results (Supplementary Table S8).

### Associations between the month of delivery and VTE during hospitalization

**Figure 2:**
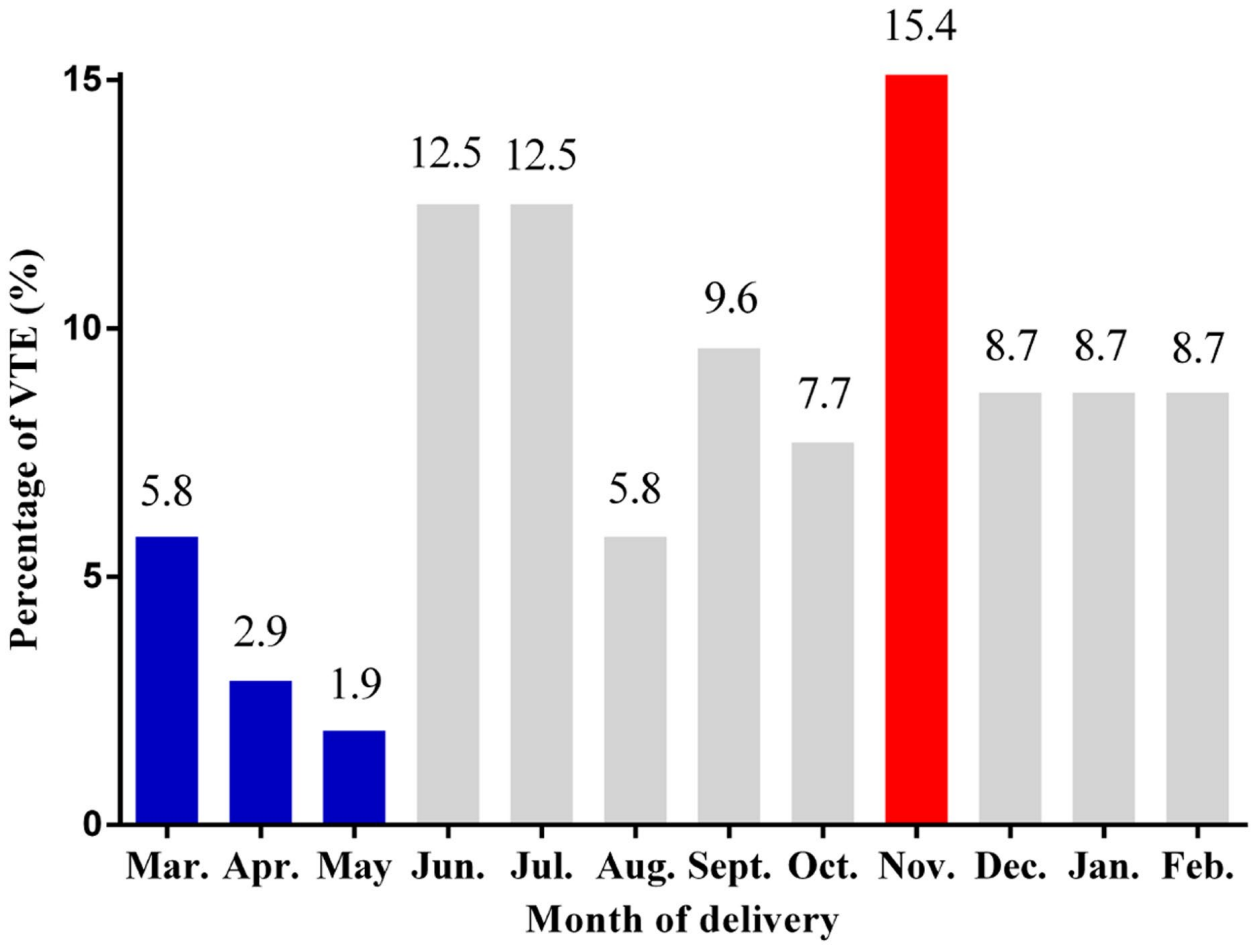
Percentages of VTE events according to the month of delivery. VTE: venous thromboembolism.

Percentages of VTE according to the month of delivery are illustrated in Figure 2. The lowest percentages of VTE events were seen in March, April, and May, and the highest percentage of VTE events was observed in November (15.4%).

**Figure 3:**
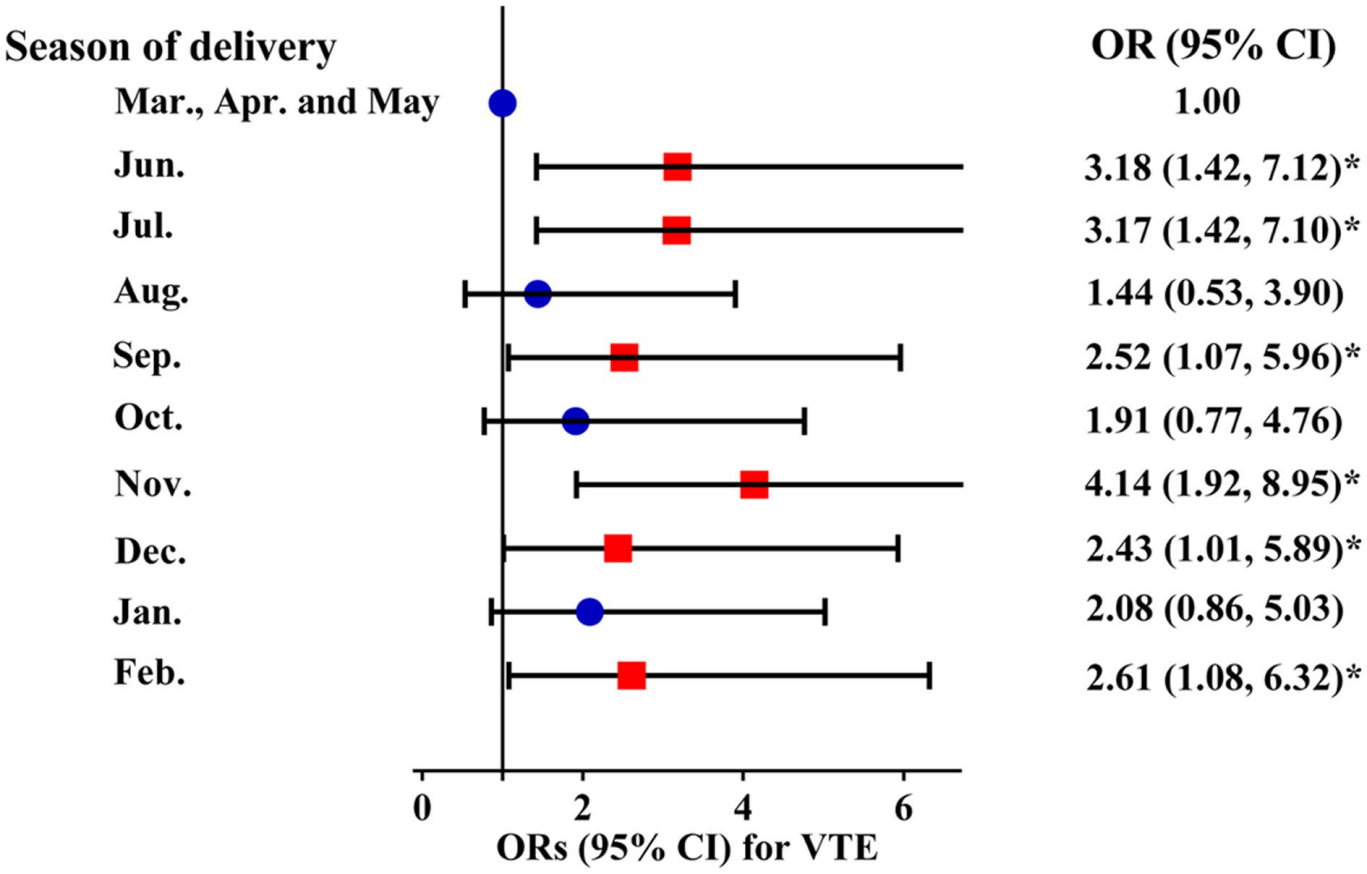
Adjusted ORs and 95% CIs for the month of delivery with VTE risk. VTE: venous thromboembolism.

Compared with participants who delivered in March, April, and May, participants who delivered in June (OR 3.18 [1.42, 7.12]), July (OR 3.17 [1.42, 7.10]), September (OR 2.52 [1.07, 5.96]), November (OR 4.14 [1.92, 8.95]), December (OR 2.43 [1.01, 5.89]), and February (OR 2.61 [1.08, 6.32]) had a higher risk of VTE during hospitalization (Figure 3).

## Discussion

To the best of our knowledge, this is the first study to report the seasonal variations of VTE in pregnant women. This study demonstrated that pregnant women who delivered in the summer, autumn, and winter had a significantly increased risk of VTE during hospitalization compared with those who delivered in spring. IVF, GDM, and preterm birth were risk factors for VTE. We adjusted for these covariates in the logistic regression models and restricted the analyses among pregnant women without IVF, GDM, and preterm, and the results still remained robust. Compared with pregnant women who delivered in March, April, and May, those who delivered in June, July, September, November, December, and February had a higher risk of VTE during hospitalization.

The occurrences of CVD have been found to be seasonal. The relationships between the seasons and the occurrences of VTE in hospitalized patients had been reported by some studies, although the conclusions were not completely consistent. Most studies found that hospitalized patients had the highest incidence of VTE in winter, (9–17) and few studies found the highest incidence of VTE in spring/summer (20–22) or no seasonality (23). Our results are similar to those conducted in Italy, Turkey, Israel, and the United States. The MASTER Registry in 25 Italian hospitals observed that VTE was most frequent in autumn (32.9%) and less frequent in spring (19%) (18). A cross-sectional study in Turkey found that the highest number of PE cases were seen in autumn (29.8%) (19). Results from the Italian Ministry of Health showed that PE hospital admissions increased by 1.07 and 0.96% in autumn and winter, respectively (17). A study from Northern Israel demonstrated that DVT and/or PE cases were most frequent in November and least frequent in May (28) which was consistent with our results. Results from East Birmingham Hospital showed that the incidence of PE in autumn was significantly higher than in the rest of the year (29).

Of the studies mentioned above, three were conducted in China. Li et al. found a winter peak in DVT patients in Shenyang (13). The lower the ambient temperature, the higher the incidence of DVT. Tan et al. suggested that hospitalized patients in Beijing were more likely to have PE in winter than in other seasons (14). A study by Lee et al. found that the percentages of VTE peaked in summer and troughed in spring (23). However, it represented non-significant seasonal differences in VTE risk in hospitalized patients in Taiwan (23). Of the three studies, the climates in the first two (Shenyang: temperate and semihumid continental climate; Beijing: temperate monsoon climate) were significantly different from that in the present study (subtropical monsoon climate), while the climate in the third study (subtropical monsoon climate/tropical monsoon climate) was similar to that in the present study. The sample size was smaller than ours (theirs: 2774; ours: 37778), which may make it difficult to observe significant differences. The current study is the only one performed on a pregnant population. It found that pregnant women who delivered in autumn had the highest incidences of VTE during hospitalization. Pregnant women who delivered in summer and winter also had an increased risk of VTE during hospitalization.

We hypothesized that our discordance with other studies may be related to the variance of the study population (pregnant women and hospitalized patients), meteorological factors (e.g., altitude, temperature, air pressure, and humidity), definitions of seasons, outcomes (VTE/DVT/PE), ethnic (genetic factors), sample size (previous studies had sample sizes of <2,800), and number of designated centers (single center and multi-center). The participants in this study are all from Hubei Province, China; therefore, we should be cautious when extrapolating the current findings to populations in other countries and regions.

The mechanisms underlying the increased risk of VTE during hospitalization among pregnant women who delivered in summer, autumn, and winter remain unclear. Pollution, climatological variables, and biological programming during pregnancy may explain the results. Participants may be more susceptible to VTE after air pollution exposure (e.g., PM_2.5_) (30). Thermoregulatory arteriovenous shunt vasoconstriction was found to promote DVT by generating venous stasis and hypoxia (31). The increased risk of VTE in summer may be related to increased temperature (32) and dehydration. Dehydration was found to be a major risk factor for VTE in human (33) and animal studies (34). The increased risk of VTE in winter may be explained by the cold temperature, the increase in blood viscosity (21), a hypercoagulable state that was ascribed to elevated fibrinogen levels (35), peripheral vasoconstriction, and the limited physical activity due to the colder temperature (36). A study among mice found that cold exposure (4°C air temperature) could reduce serum adiponectin, which may help in protection against arteriosclerosis (37). In this study, participants who delivered in autumn yielded the highest VTE risk compared to winter or summer. Given that the temperatures in autumn and spring were similar, we hypothesize that the increased risk of VTE in autumn may be associated with the rapid change in temperature and air pressure in autumn (38). The exact mechanisms are still unclear, and more research should be carried out in the future.

Pregnant women with IVF pregnancy have an increased risk of VTE due to increased estrogen levels that lead to hypercoagulability (7). GDM increases the risk of VTE, which may be related to the vascular inflammation promoted by high blood glucose (39). Preterm delivery was observed to be a conventional pregnancy-associated VTE risk factor (40). In this study, potential confounders of IVF pregnancy, GDM, and preterm were adjusted and considered in sensitivity analyses. The main results were still stable, indicating that the associations between the season of delivery and VTE during hospitalization were independent of the covariates mentioned above.

Our study has several strengths that are worth mentioning. First, this is the first study to report an increased risk of VTE occurring in pregnant women who delivered in other seasons compared with those who delivered in spring. Second, the sample sizes of previous studies on the seasonal occurrences of VTE were less than 2,800, and the sample size of this study was the largest. Most of the previous studies were single-center studies, whereas this study is a multi-center study, thus the reliability of the study results increased. Third, this study has the ability to exclude those who had VTE or medication thromboprophylaxis within 2 weeks of admission, which clarified the results. Fourth, all the data in this study were diagnosed and analyzed by two researchers independently. If there was any inconsistency, a third researcher would participate to make the data more reliable. This study had comprehensive covariates including demographic, lifestyle, history of reproduction, and disease, allowing for adjustment.

This study has several limitations. First, certain women may possess a genetic inclination toward the development of VTE, which is referred to as hereditary thrombophilia. Hereditary factors were not considered in this study, though participants with a history of prenatal VTE were excluded. Second, retrospective data collection may be prone to missing data, measurement errors, and inaccuracy. Pregnant women who were overweight or obese before pregnancy had an increased risk of VTE. Although this study included as many covariates as possible, pre-pregnancy body mass index was not considered. Subsequent studies should include the covariate of pre-pregnancy body mass index. Third, this study only discussed the relationships between season and VTE. The relationships between meteorological factors (e.g., temperature and humidity) and VTE remain to be investigated in the future. Finally, the rates of VTE vary greatly between Chinese and foreign populations. The participants of this study were all from Hubei Province, China, a tropical monsoon climate region. Therefore, caution is required when extrapolating the conclusions of this study to populations in other countries and regions.

## Conclusion

In conclusion, this is the first study demonstrating that pregnant women who delivered in summer, autumn, and winter had a significantly increased risk of VTE during hospitalization compared with those who delivered in spring. This study suggests that VTE risk assessment and thromboprophylaxis should be strengthened in pregnant women, especially for those who delivered in summer, autumn, and winter, which might have important public health implications in the prevention of VTE. More multi-center and mechanistic studies are warranted in the future.

## Data availability statement

The original contributions presented in the study are included in the article/Supplementary material, further inquiries can be directed to the corresponding authors.

## Ethics statement

The studies involving humans were approved by Tongji Medical College affiliated with Huazhong University of Science and Technology. The studies were conducted in accordance with the local legislation and institutional requirements. Written informed consent for participation was not required from the participants or the participants' legal guardians/next of kin because the information was retrieved from the medical records retrospectively.

## Author contributions

QL: Conceptualization, Data curation, Formal analysis, Funding acquisition, Methodology, Project administration, Validation, Visualization, Writing – original draft, Writing – review & editing. HoW: Conceptualization, Data curation, Formal analysis, Validation, Writing – review & editing. HuW: Formal analysis, Validation, Writing – review & editing. JD: Formal analysis, Validation, Writing – review & editing. ZC: Formal analysis, Validation, Writing – review & editing. WL: Formal analysis, Validation, Writing – review & editing. RZ: Formal analysis, Validation, Writing – review & editing. SC: Formal analysis, Validation, Writing – review & editing. JG: Formal analysis, Validation, Writing – review & editing. HL: Formal analysis, Validation, Writing – review & editing. YC: Formal analysis, Validation, Writing – review & editing. XY: Formal analysis, Validation, Writing – review & editing. SD: Formal analysis, Validation, Writing – review & editing. YT: Formal analysis, Validation, Writing – review & editing. YX: Formal analysis, Validation, Writing – review & editing. PW: Formal analysis, Validation, Writing – review & editing. FZ: Formal analysis, Validation, Writing – review & editing. XW: Formal analysis, Validation, Writing – review & editing. LT: Conceptualization, Data curation, Formal analysis, Funding acquisition, Methodology, Project administration, Validation, Writing – original draft, Writing – review & editing. YH: Conceptualization, Data curation, Formal analysis, Methodology, Project administration, Validation, Visualization, Writing – original draft, Writing – review & editing.

## References

[1] Jang MJ Bang SM Oh D. Incidence of pregnancy-associated venous thromboembolism in Korea: from the Health Insurance Review and Assessment Service database. J Thromb Haemost. (2011) 9:2519–21. doi: 10.1111/j.1538-7836.2011.04518.x, PMID: 21951997

[2] Heit JA Kobbervig CE James AH Petterson TM Bailey KR Melton LJ 3rd. Trends in the incidence of venous thromboembolism during pregnancy or postpartum: a 30-year population-based study. Ann Intern Med. (2005) 143:697–706. doi: 10.7326/0003-4819-143-10-200511150-00006, PMID: 16287790

[3] Kourlaba G Relakis J Kontodimas S Holm MV Maniadakis N. A systematic review and meta-analysis of the epidemiology and burden of venous thromboembolism among pregnant women. Int J Gynaecol Obstet. (2016) 132:4–10. doi: 10.1016/j.ijgo.2015.06.054 26489486

[4] Holley AB. Preventing venous thromboembolism without causing harm. Lancet Reg Health West Pac. (2021) 6:100066. doi: 10.1016/j.lanwpc.2020.100066, PMID: 34327402 PMC8315367

[5] Nichols KM Henkin S Creager MA. Venous thromboembolism associated with pregnancy: JACC focus seminar. J Am Coll Cardiol. (2020) 76:2128–41. doi: 10.1016/j.jacc.2020.06.090 33121721

[6] Rodger M. Pregnancy and venous thromboembolism: 'TIPPS' for risk stratification. Hematology Am Soc Hematol Educ Program. (2014) 2014:387–92. doi: 10.1182/asheducation-2014.1.387, PMID: 25696883

[7] Sennström M Rova K Hellgren M Hjertberg R Nord E Thurn L. Thromboembolism and in vitro fertilization - a systematic review. Acta Obstet Gynecol Scand. (2017) 96:1045–52. doi: 10.1111/aogs.13147, PMID: 28382684

[8] Marti-Soler H Gonseth S Gubelmann C Stringhini S Bovet P Chen PC. Seasonal variation of overall and cardiovascular mortality: a study in 19 countries from different geographic locations. PLoS One. (2014) 9:e113500. doi: 10.1371/journal.pone.0113500, PMID: 25419711 PMC4242652

[9] Fink AM Mayer W Steiner A. Seasonal variations of deep vein thrombosis and its influence on the location of the thrombus. Thromb Res. (2002) 106:97–100. doi: 10.1016/S0049-3848(02)00094-4, PMID: 12182906

[10] Gallerani M Boari B Smolensky MH Salmi R Fabbri D Contato E. Seasonal variation in occurrence of pulmonary embolism: analysis of the database of the EmiliaRomagna region. Italy Chronobiol Int. (2007) 24:143–60. doi: 10.1080/07420520601139755 17364585

[11] Ribeiro DD Bucciarelli P Braekkan SK Lijfering WM Passamonti SM Brodin EE. Seasonal variation of venous thrombosis: a consecutive case series within studies from Leiden. Milan and Tromso J Thromb Haemost. (2012) 10:1704–7. doi: 10.1111/j.1538-7836.2012.04811.x 22681473

[12] Skajaa N Horváth-Puhó E Adelborg K Prandoni P Rothman KJ Sørensen HT. Venous thromboembolism in Denmark: seasonality in occurrence and mortality. TH Open. (2019) 3:e171–9. doi: 10.1055/s-0039-1692399 31259300 PMC6598086

[13] Li Y Ji C Ju H Han Y. Impact of ambient temperature and atmospheric evaporation on the incidence of acute deep venous thrombosis in the northeast of China. Int Angiol. (2017) 36:243–53. doi: 10.23736/S0392-9590.16.03730-5, PMID: 27575331

[14] Tan XY He JG Zou ZP Zhao YF Chen BP Gao Y. Changes of the proportion and mortality of pulmonary thromboembolism in hospitalized patients from 1974 to 2005. Chin Med J. (2006) 119:998–1002. doi: 10.1097/00029330-200606020-00006, PMID: 16805983

[15] Dentali F Manfredini R Ageno W. Seasonal variability of venous thromboembolism. Curr Opin Pulm Med. (2009) 15:403–7. doi: 10.1097/MCP.0b013e32832d867a, PMID: 19542893

[16] Zhao H Li Y Wu M Ren W Ji C Miao H. Seasonal variation in the frequency of venous thromboembolism: an updated result of a meta-analysis and systemic review. Phlebology. (2020) 35:480–94. doi: 10.1177/0268355519897650, PMID: 32036737

[17] Di Blasi C Renzi M Michelozzi P Scortichini M Davoli M Forastiere F. Association between air temperature, air pollution and hospital admissions for pulmonary embolism and venous thrombosis in Italy. Eur J Intern Med. (2022) 96:74–80. doi: 10.1016/j.ejim.2021.09.019, PMID: 34702659

[18] Manfredini R Imberti D Gallerani M. Seasonal variation in the occurrence of venous thromboembolism: data from the MASTER registry. Clin Appl Thromb Hemost. (2009) 15:309–15. doi: 10.1177/1076029608319947, PMID: 18544594

[19] Anar C Inal T Erol S. Are meteorological parameters a risk factor for pulmonary embolism? A retrospective analysis of 530 patients. Balkan Med J. (2015) 32:279–84. doi: 10.5152/balkanmedj.2015.15686, PMID: 26185716 PMC4497694

[20] Chiu HH Whittaker P. Venous thromboembolism in an industrial north american city: temporal distribution and association with particulate matter air pollution. PLoS One. (2013) 8:e68829. doi: 10.1371/journal.pone.0068829, PMID: 23874781 PMC3707887

[21] Oztuna F Ozsu S Topbas M Bulbul Y Kosucu P Ozlu T. Meteorological parameters and seasonal variations in pulmonary thromboembolism. Am J Emerg Med. (2008) 26:1035–41. doi: 10.1016/j.ajem.2007.12.010, PMID: 19091266

[22] Meral M Mirici A Aslan S Akgun M Kaynar H Saglam L. Barometric pressure and the incidence of pulmonary embolism. Chest. (2005) 128:2190–4. doi: 10.1378/chest.128.4.2190, PMID: 16236873

[23] Lee CH Cheng CL Lin LJ Tsai LM Yang YH. Epidemiology and predictors of short-term mortality in symptomatic venous thromboembolism. Circ J. (2011) 75:1998–2004. doi: 10.1253/circj.CJ-10-0992, PMID: 21697611

[24] Roy PM Barbeau C Ternisien C Leftheriotis G. Diagnostic strategy in deep-vein thrombosis. Lancet. (1998) 351:1588. doi: 10.1016/S0140-6736(05)61158-0, PMID: 10348672

[25] Perrier A Bounameaux H Morabia A de Moerloose P Slosman D Didier D. Diagnosis of pulmonary embolism by a decision analysis-based strategy including clinical probability, D-dimer levels, and ultrasonography: a management study. Arch Intern Med. (1996) 156:531–6. doi: 10.1001/archinte.1996.00440050079009, PMID: 8604959

[26] American Diabetes Association. Diagnosis and classification of diabetes mellitus. Diabetes Care. (2014) 37:S81–90. doi: 10.2337/dc14-S081 24357215

[27] Lo JO Mission JF Caughey AB. Hypertensive disease of pregnancy and maternal mortality. Curr Opin Obstet Gynecol. (2013) 25:124–32. doi: 10.1097/GCO.0b013e32835e0ef5, PMID: 23403779

[28] Elias S Hoffman R Saharov G Brenner B Nadir Y. Dehydration as a possible cause of monthly variation in the incidence of venous thromboembolism. Clin Appl Thromb Hemost. (2016) 22:569–74. doi: 10.1177/1076029616649435, PMID: 27206642

[29] Green J Edwards C. Seasonal variation in the necropsy incidence of massive pulmonary embolism. J Clin Pathol. (1994) 47:58–60. doi: 10.1136/jcp.47.1.58, PMID: 8132811 PMC501758

[30] Dales RE Cakmak S Vidal CB. Air pollution and hospitalization for venous thromboembolic disease in Chile. J Thromb Haemost. (2010) 8:669–74. doi: 10.1111/j.1538-7836.2010.03760.x, PMID: 20088925

[31] Tayefeh F Kurz A Sessler DI Lawson CA Ikeda T Marder D. Thermoregulatory vasodilation increases the venous partial pressure of oxygen. Anesth Analg. (1997) 85:657–62. doi: 10.1213/00000539-199709000-00031, PMID: 9296426

[32] Nimako K Poloniecki J Draper A Rahman T. Seasonal variability and meteorological factors: retrospective study of the incidence of pulmonary embolism from a large United Kingdom teaching hospital. Respir Care. (2012) 57:1267–72. doi: 10.4187/respcare.01129, PMID: 22348516

[33] Jackson BF Porcher FK Zapton DT Losek JD. Cerebral sinovenous thrombosis in children: diagnosis and treatment. Pediatr Emerg Care. (2011) 27:874–80. doi: 10.1097/PEC.0b013e31822c9ccc, PMID: 21926891

[34] Tanira MO Sabban FF Fahim MA Wasfi IA. Acetyl salicylic acid alleviates increased susceptibility to thrombosis in pial microvessels of dehydrated mice. J Vet Med Sci. (1994) 56:245–8. doi: 10.1292/jvms.56.245, PMID: 8075211

[35] Manfredini R Gallerani M Salmi R Dentali F Ageno W. Winter and venous thromboembolism: a dangerous liaison? Futur Cardiol. (2011) 7:717–9. doi: 10.2217/fca.11.61, PMID: 22050054

[36] Sumukadas D Witham M Struthers A McMurdo M. Day length and weather conditions profoundly affect physical activity levels in older functionally impaired people. J Epidemiol Community Health. (2009) 63:305–9. doi: 10.1136/jech.2008.080838, PMID: 19074181

[37] Imai J Katagiri H Yamada T Ishigaki Y Ogihara T Uno K. Cold exposure suppresses serum adiponectin levels through sympathetic nerve activation in mice. Obesity. (2006) 14:1132–41. doi: 10.1038/oby.2006.130, PMID: 16899794

[38] Törő K Pongrácz R Bartholy J Váradi-t A Marcsa B Szilágyi B. Evaluation of meteorological and epidemiological characteristics of fatal pulmonary embolism. Int J Biometeorol. (2016) 60:351–9. doi: 10.1007/s00484-015-1032-8, PMID: 26178756

[39] Jacobsen AF Skjeldestad FE Sandset PM. Incidence and risk patterns of venous thromboembolism in pregnancy and puerperium–a register-based case-control study. Am J Obstet Gynecol. (2008) 198(2):233:e231–7. doi: 10.1016/j.ajog.2007.08.041 17997389

[40] Jaya-Bodestyne SL Lee LH Tan LK Tan KH Østbye T Malhotra R. Risk factors for pregnancy-associated venous thromboembolism in Singapore. J Perinat Med. (2021) 49:153–8. doi: 10.1515/jpm-2020-0298, PMID: 32889795

