# Supplementary material for: Season of delivery and risk of venous thromboembolism during hospitalization among pregnant women
Source: Front Public Health. 2023 Nov 8;11:1272149. doi: 10.3389/fpubh.2023.1272149 (PMC10663352; doi:10.3389/fpubh.2023.1272149)
Supplement: Supplementary file 1 [file Table_1.doc]

**Title:** Season of delivery and risk of venous thromboembolism during hospitalization among pregnant women

**Table S1—The seven designated sites in this study**

| **No.** | **Name of the designated site** | **City** | **Location** |
| --- | --- | --- | --- |
| **1** | Union Hospital, Tongji Medical College, Huazhong University of Science and Technology (Main Campus) | Wuhan | Hubei Province, China |
| **2** | Union Hospital, Tongji Medical College, Huazhong University of Science and Technology (West Campus) | Wuhan | Hubei Province, China |
| **3** | Union Hospital, Tongji Medical College, Huazhong University of Science and Technology (Cancer Centre) | Wuhan | Hubei Province, China |
| **4** | Jingshan Union Hospital, Union Hospital, Huazhong University of Science and Technology | Jingshan | Hubei Province, China |
| **5** | People's Hospital of Dongxihu District | Wuhan | Hubei Province, China |
| **6** | Central Hospital of Hefeng County | Enshi | Hubei Province, China |
| **7** | The Sixth Hospital of Wuhan | Wuhan | Hubei Province, China |

**Table S2—Characteristics of the participants included and excluded from this study**

| **Characteristics** | **Total**  **(n = 37908)** | **Included**  **(n = 37778)** | **Excluded**  **(n = 130)** |
| --- | --- | --- | --- |
| **Maternal age at delivery (years)** | 29.9 ± 4.6 | 29.9 ± 4.6 | 29.5 ± 4.7 |
| **Multiple pregnancy (%)** | 938 (2.5) | 934 (2.5) | 4 (3.1) |
| **Primipara (%)** | 23655 (62.4) | 23562 (62.4) | 93 (71.5) |
| **Habit of drinking (%)** | 21 (0.06) | 21 (0.06) | 0 (0.00) |
| **Habit of smoking (%)** | 126 (0.3) | 126 (0.3) | 0 (0.00) |

**Table S3—Characteristics of the participants before and after matching**

| **Characteristics** | **Before matching** | |  |  | **After matching** | |  |
| --- | --- | --- | --- | --- | --- | --- | --- |
|  | **Spring**  **(n=9191)** | **Non-spring**  **(n=28587)** | ***P*** |  | **Spring**  **(n=9188)** | **Non-spring**  **(n=9188)** | ***P*** |
| **Maternal age at delivery (years)** | 29.9 ± 4.6 | 29.9 ± 4.6 | 0.955 |  | 29.9 ± 4.6 | 29.9 ± 4.6 | 0.650 |
| **IVF pregnancy (%)** | 411 (4.5) | 1301 (4.6) | 0.772 |  | 411 (4.5) | 400 (4.4) | 0.719 |
| **Multiple pregnancy (%)** | 242 (2.6) | 692 (2.4) | 0.263 |  | 241 (2.6) | 217 (2.4) | 0.276 |
| **Primipara (%)** | 5788 (63.0) | 17774 (62.2) | 0.170 |  | 5786 (63.0) | 5775 (62.9) | 0.867 |
| **Habit of drinking (%)** | 4 (0.04) | 17 (0.1) | 0.799 |  | 1 (0.01) | 1 (0.01) | 0.625 |
| **Habit of smoking (%)** | 35 (0.4) | 91 (0.3) | 0.353 |  | 34 (0.4) | 31 (0.3) | 0.804 |
| **History of diabetes (%)** | 21 (0.2) | 63 (0.2) | 0.907 |  | 21 (0.2) | 19 (0.2) | 0.874 |
| **GDM (%)** | 961 (10.5) | 3572 (12.5) | <0.001 |  | 961 (10.5) | 943 (10.3) | 0.681 |
| **Preeclampsia (%)** | 263 (2.9) | 831 (2.9) | 0.856 |  | 263 (2.9) | 244 (2.7) | 0.418 |
| **Preterm (%)** | 461 (5.0) | 1572 (5.5) | 0.076 |  | 461 (5.0) | 450 (4.9) | 0.734 |

Abbreviations: GDM: gestational diabetes mellitus; IVF: *in vitro* fertilization.

The comparison of “maternal age at delivery” was performed with t test. The comparison of categorical variables among the four groups was performed with chi-square test.

**P* <0.05.

**Table S4—Adjusted ORs and 95% CIs for season of delivery and VTE risk during hospitalization after matching**

|  | **Spring**  **(n=9188)** | **Non-spring**  **(n=9188)** |
| --- | --- | --- |
| **No. of VTE (%)** | 11 (0.12%) | 25 (0.27%) |
| **Unadjusted** | 1 | 2.28 (1.12, 4.62) |
| **Model 1** | 1 | 2.29 (1.13, 4.66) |
| **Model 2** | 1 | 2.30 (1.13, 4.69) |

Abbreviations: CI: confidence interval; OR: odds ratio; VTE: venous thromboembolism.

Model 1 adjusted for maternal age at delivery, *in vitro* fertilization pregnancy, multiple pregnancy, and primipara.

Model 2 adjusted for habit of drinking, habit of smoking, history of diabetes, gestational diabetes mellitus, preeclampsia, preterm, postpartum hemorrhage, delivery mode and covariates included in model 1.

**Table S5—Adjusted ORs and 95% CIs for season of delivery and VTE risk during hospitalization excluding pregnant women with IVF pregnancy (n=36066)**

|  | **Season of delivery** | | | |  | |
| --- | --- | --- | --- | --- | --- | --- |
|  | **Spring**  **(n=8780)** | **Summer**  **(n=9302)** | **Autumn**  **(n=9147)** | **Winter**  **(n=8837)** |  | **Combined (summer + autumn + winter)†**  **(n=27286)** |
| **No. of VTE (%)** | 10 (0.11%) | 29 (0.31%) | 32 (0.35%) | 23 (0.26%) |  | 84 (0.31%) |
| **Unadjusted** | 1 | 2.74 (1.34, 5.63) | 3.08 (1.51, 6.27) | 2.29 (1.09, 4.81) |  | 2.71 (1.41, 5.22) |
| **Model 1** | 1 | 2.70 (1.32, 5.55) | 3.07 (1.51, 6.25) | 2.29 (1.09, 4.83) |  | 2.69 (1.40, 5.19) |
| **Model 2** | 1 | 2.63 (1.28, 5.40) | 2.92 (1.43, 5.95) | 2.21 (1.05, 4.65) |  | 2.59 (1.34, 5.00) |

Abbreviations: CI: confidence interval; OR: odds ratio; VTE: venous thromboembolism.

Model 1 adjusted for maternal age at delivery, multiple pregnancy, and primipara.

Model 2 adjusted for habit of drinking, habit of smoking, history of diabetes, gestational diabetes mellitus, preeclampsia, preterm, postpartum hemorrhage, delivery mode and covariates included in model 1.

†The reference group is spring.

**Table S6—Adjusted ORs and 95% CIs for season of delivery and VTE risk during hospitalization excluding pregnant women with GDM (n=33245)**

|  | **Season of delivery** | | | |  | |
| --- | --- | --- | --- | --- | --- | --- |
|  | **Spring**  **(n=8230)** | **Summer**  **(n=8583)** | **Autumn**  **(n=8263)** | **Winter**  **(n=8169)** |  | **Combined (summer + autumn + winter)** **†**  **(n=25015)** |
| **No. of VTE (%)** | 8 (0.10%) | 23 (0.27%) | 25 (0.30%) | 19 (0.23%) |  | 67 (0.27%) |
| **Unadjusted** | 1 | 2.88 (1.29, 6.42) | 3.12 (1.41, 6.92) | 2.40 (1.05, 5.48) |  | 2.80 (1.35, 5.83) |
| **Model 1** | 1 | 2.86 (1.28, 6.37) | 3.15 (1.42, 7.00) | 2.41 (1.05, 5.50) |  | 2.81 (1.35, 5.84) |
| **Model 2** | 1 | 2.74 (1.23, 6.11) | 3.09 (1.39, 6.85) | 2.36 (1.03, 5.39) |  | 2.73 (1.31, 5.68) |

Abbreviations: CI: confidence interval; OR: odds ratio; VTE: venous thromboembolism.

Model 1 adjusted for maternal age at delivery, *in vitro* fertilization pregnancy, multiple pregnancy, and primipara.

Model 2 adjusted for habit of drinking, habit of smoking, history of diabetes, preeclampsia, preterm, postpartum hemorrhage, delivery mode and covariates included in model 1.

†The reference group is spring.

**Table S7—Adjusted ORs and 95% CIs for season of delivery and VTE risk during hospitalization excluding pregnant women with preterm (n=35745)**

|  | **Season of delivery** | | | |  | |
| --- | --- | --- | --- | --- | --- | --- |
|  | **Spring**  **(n=8730)** | **Summer**  **(n=9196)** | **Autumn**  **(n=9020)** | **Winter**  **(n=8799)** |  | **Combined (summer + autumn + winter)** **†**  **(n=27015)** |
| **No. of VTE (%)** | 7 (0.08%) | 28 (0.30%) | 31 (0.34%) | 27 (0.31%) |  | 86 (0.32%) |
| **Unadjusted** | 1 | 3.81 (1.66, 8.72) | 4.30 (1.89, 9.76) | 3.84 (1.67, 8.81) |  | 3.98 (1.84, 8.60) |
| **Model 1** | 1 | 3.77 (1.64, 8.63) | 4.33 (1.90, 9.83) | 3.83 (1.67, 8.80) |  | 3.97 (1.84, 8.59) |
| **Model 2** | 1 | 3.66 (1.60, 8.40) | 4.12 (1.81, 9.38) | 3.68 (1.60, 8.47) |  | 3.82 (1.77, 8.27) |

Abbreviations: CI: confidence interval; OR: odds ratio; VTE: venous thromboembolism.

Model 1 adjusted for maternal age at delivery, *in vitro* fertilization pregnancy, multiple pregnancy, and primipara.

Model 2 adjusted for habit of drinking, habit of smoking, history of diabetes, gestational diabetes mellitus, preeclampsia, postpartum hemorrhage, delivery mode and covariates included in model 1.

†The reference group is spring.

**Table S8—Adjusted ORs and 95% CIs for season of delivery and VTE risk during hospitalization for delivery excluding participants enrolled in 2020 (n=33216)**

|  | **Season of delivery** | | | |  | |
| --- | --- | --- | --- | --- | --- | --- |
|  | **Spring**  **(n=8290)** | **Summer**  **(n=8349)** | **Autumn**  **(n=8363)** | **Winter**  **(n=8164)** |  | **Combined (summer + autumn + winter)†**  **(n=24926)** |
| **No. of VTE (%)** | 11 (0.13%) | 28 (0.34%) | 27 (0.32%) | 26 (0.32%) |  | 81 (0.32%) |
| **Unadjusted** | 1 | 2.52 (1.25, 5.06) | 2.44 (1.21, 4.92) | 2.41 (1.19, 4.87) |  | 2.45 (1.31, 4.61) |
| **Model 1** | 1 | 2.49 (1.24, 5.00) | 2.45 (1.21, 4.94) | 2.41 (1.19, 4.88) |  | 2.45 (1.30, 4.60) |
| **Model 2** | 1 | 2.43 (1.21, 4.89) | 2.36 (1.17, 4.77) | 2.34 (1.15, 4.74) |  | 2.38 (1.27, 4.47) |

Abbreviations: CI: confidence interval; OR: odds ratio; VTE: venous thromboembolism.

Model 1 adjusted for maternal age at delivery, *in vitro* fertilization pregnancy, multiple pregnancy, and primipara.

Model 2 adjusted for habit of drinking, habit of smoking, history of diabetes, gestational diabetes mellitus, preeclampsia, preterm, postpartum hemorrhage, delivery mode and covariates included in model 1.

†The reference group is spring.

37778 pregnant women

37908 pregnant women who delivered

in 7 centers in Hubei Province, China

between January, 2017 and December, 2022

Excluded pregnant women with previous VTE at admission, or medication thromboprophylaxis within 2 weeks at admission (n=130)

Delivered in spring (n=9191)

Delivered in summer (n=9808)

Delivered in autumn (n=9550)

Delivered in winter (n=9229)

**Figure S1——Flow diagram of this study**

Abbreviations: VTE: venous thromboembolism.
